# Human embryonal epithelial cells of the developing small intestinal crypts can express the Hodgkin-cell associated antigen Ki-1 (CD30) in spontaneous abortions during the first trimester of gestation

**DOI:** 10.1186/1742-4682-2-1

**Published:** 2005-01-11

**Authors:** Demetrio Tamiolakis, John Venizelos, Maria Lambropoulou, Silva Nikolaidou, Sophia Bolioti, Maria Tsiapali, Dionysios Verettas, Panagiotis Tsikouras, Athanasios Chatzimichail, Nikolas Papadopoulos

**Affiliations:** 1 Department of Cytology, General Hospital of Chania, Crete, Greece; 2 Department of Pathology, Ippokration Hospital of Salonica, Greece; 3 Department of Histology – Embryology, Democritus University of Thrace, Greece; 4 Department of Obstetrics and Gynecology, Democritus University of Thrace, Greece

**Keywords:** CD30 (Ki-1) antigen, human intestinal cells, spontaneous abortions, voluntary or therapeutic abortions, first trimester of gestation.

## Abstract

**Background:**

Ki-1 (CD30) antigen expression is not found on peripheral blood cells but its expression can be induced in vitro on T and B lymphocytes by viruses and lectins. Expression of CD30 in normal tissues is very limited, being restricted mainly to a subpopulation of large lymphoid cells; in particular, cells of the recently described anaplastic large cell lymphoma (ALCL), the Reed-Sternberg (RS) cells of Hodgkin's lymphoma and scattered large parafollicular cells in normal lymphoid tissues. More recent reports have described CD30 expression in non-hematopoietic and malignant cells such as cultured human macrophages, human decidual cells, histiocytic neoplastic cells, mesothelioma cells, embryonal carcinoma and seminoma cells.

**Results:**

We investigated the immunohistochemical expression of CD30 antigen in 15 paraffin-embedded tissue samples representing small intestines from fetuses after spontaneous abortion in the 8th, 10th and 12th weeks using the monoclonal antibody Ki-1. Hormones had been administered to all our pregnant women to support gestation. In addition, a panel of monoclonal antibodies was used to identify leukocytes (CD45/LCA), B-lymphocytes (CD20/L-26) and T-lymphocytes (CD3). Our findings were correlated with those obtained simultaneously from intestinal tissue samples obtained from 15 fetuses after therapeutic or voluntary abortions.

**Conclusions:**

The results showed that: (1) epithelial cells in the developing intestinal crypts express the CD30 (Ki-1) antigen; (2) CD30 expression in these epithelial cells is higher in cases of hormonal administration than in normal gestation. In the former cases (hormonal support of gestation) a mild mononuclear intraepithelial infiltrate composed of CD3 (T-marker)-positive cells accompanies the CD30-positive cells.

## Introduction

CD30 antigen, a member of the tumor necrosis factor (TNF) receptor superfamily [1-3], was originally identified as a cell surface antigen on primary and cultured Hodgkin's and Reed-Sternberg cells by use of the monoclonal antibody Ki-1 [4,5]. CD30 antigen is normally expressed by a subset (15–20%) of CD3+ T cells after activation by various stimuli [6]. Its expression is stimulated by interleukin (IL)-4 during lineage commitment of naïve human T cells [7,8] and is augmented by the presence of CD28 co-stimulatory signals [9]. CD30 also is expressed at variable levels in different non-Hodgkin's lymphomas (NHL) as well as in several virally transformed T and B cell lines [5,10]. In particular, CD30 is a specific marker of a subset of peripheral T cell NHLs known as anaplastic large cell lymphomas (ALCL) [5]. More recently, preferential CD30 expression has been detected on a subset of tissue and circulating CD4+ and CD8+ T cells producing mainly Th2 cytokines in immunoreactive conditions [11-14].

CD30 appears to have an important immunoregulatory role in normal T cell development. Within the thymus, CD30L is highly expressed on medullary thymic epithelial cells and on Hassal's corpuscles [15].

Pallesen and Hamilton-Dutoir [16] were the first to report CD30 expression outside lymphoid tissue in 12 out of 14 cases of primary or metastatic embryonal carcinoma (EC) of the testis, using immunostaining with the monoclonal antibodies (MAbs) Ber-H2 and Ki-1. Subsequently, several investigators have confirmed their results and have detected CD30 in these carcinomas at the protein [17-20] and the mRNA [10] level. Two reports demonstrated CD30 expression in 4/21 and 4/63 cases of testicular and mediastinal seminoma [21] and in the seminomatous components of 7/14 cases of mixed germ cell tumours of the testis [22]. Suster et al. detected the CD30 antigen in 6/25 yolk sac tumours of the testis and mediastinum [22]. CD30 expression has also been reported in other non-lymphoid tissues and cells such as soft tissue tumours [23], decidual cells [24,25], lipoblasts [26], myoepithelial cells [27], reactive and neoplastic vascular lesions [28], mesotheliomas [29], cultivated macrophages, and two histiocytic malignancies [30].

Primitive crypts (epithelial downgrowths into the mesenchyme between the small intestinal villi), appear in the postpharyngeal foregut between the 9^th ^and 12^th ^weeks of embryo development. Goblet cells are present in small numbers after 8 weeks, Paneth cells differentiate at the base of the crypts in weeks 11 and 12, and enteroendocrine cells appear between weeks 9 and 11.

The fact that the CD30 molecule can mediate signals for cell proliferation or apoptosis [2] prompted us to perform a systematic investigation of CD30 antigen expression in non-hematopoietic embryonal tissues during the proliferation and differentiation stages, beginning with the epithelial cells of the developing intestinal crypts.

## Materials and methods

Samples representing 15 small intestines from fetuses after spontaneous (involuntary) abortion occurring in pregnant women treated with progesterone (300–600 mg per day until the 12th gestational week), and 15 small intestines from fetuses after therapeutic or voluntary abortion, were obtained in the 8th, 10th and 12th weeks of gestation. The Regional Ethics Committees approved the study. Written informed consent was obtained from all individuals and the procedures followed accorded with institutional guidelines. Small intestines were cut in 3 mm slices and fixed in 10% neutral buffered formaldehyde at 4°C for 24 h, then processed for routine paraffin embedding. Paraffin blocks were available in all cases, and 3 μm thick tissue sections were stained routinely with hematoxylin-eosin, PAS and Giemsa, and subsequently by immunohistochemistry. Immunoperoxidase labeling was performed as follows: sections were deparaffinized in 70% alcohol and endogenous peroxidase was blocked with 3% H_2_O_2 _in methanol. The sections were preincubated in 20% serum of the species from which the secondary antibody was raised, and the primary antibody was applied. After overnight incubation at room temperature, the secondary biotinylated antibody was applied for 30 min. Staining was visualized with a Vector Elite System (Vector Laboratories, Burlingame, CA) using diaminobenzidine as the chromogen. The sections were counterstained with dilute hematoxylin. The primary antibodies used were as follows: (CD30/Ki-1) activated lymphoid cells, mouse monoclonal antibody (Novocastra); (CD45/LCA) leukocyte common antigen, mouse monoclonal antibody (Dako); (CD20/L-26) B-lymphocytes, mouse monoclonal antibody (Dako); and (CD3) T-lymphocytes, mouse monoclonal antibody (Dako). We used the high temperature antigen unmasking technique for immunohistochemical demonstration of CD30/Ki-1 on paraffin sections (Novocastra). Control slides were incubated with nonimmunized rabbit serum. An anaplastic lymphoma case-slide (positive control) was run in parallel with the assay.

### Analysis of CD30/Ki-1 positive cryptae cells

For each sample, the CD30/Ki-1 positive population was assessed by enumeration of labeled cells in each tissue compartment for a minimum of five random fields per section viewed at 40-fold magnification through a grid. Cell numbers were calculated per mm^2 ^of tissue section. The counted areas were selected from random tissue sections, taking into account that the ratio of the area of the intestinal stroma to the area of surface epithelium covering the crypts was representative of the entire field. Areas with obvious necrosis or haemorrhages were excluded. Statistical analysis was performed using the ANOVA test.

## Results

Five microscopic fields of the small intestines were evaluated in each case without knowledge of the clinical data (TABLE 1). Two observers examined the sections independently, and positive cellular staining for each antibody was manifested as fine brown cytoplasmic granularity and/or surface membrane expression.

**Table 1:** Expresion of CD30 antigen in fetal intestinal cryptae cells during the first trimester of gestation.

	**Spontaneous abortions**			**Voluntary abortions**		
	8th week	10th week	statistics	8th week	10th week	statistics
CD30(+)cells/mm^2^	3.61+/0.16	5.27+/-0.19	p < 0.0001	3.42+/-0.17	3.43+/-0.18	p = 0.95
	8th week	12th week	statistics	8th week	12th week	statistics
CD30(+)cells/mm^2^	3.61+/-0.16	5.34+/-0.23	p < 0.0001	3.42+/-0.17	3.41+/-0.17	p = 0.95

### 8th week of gestation

**Figure 1:**
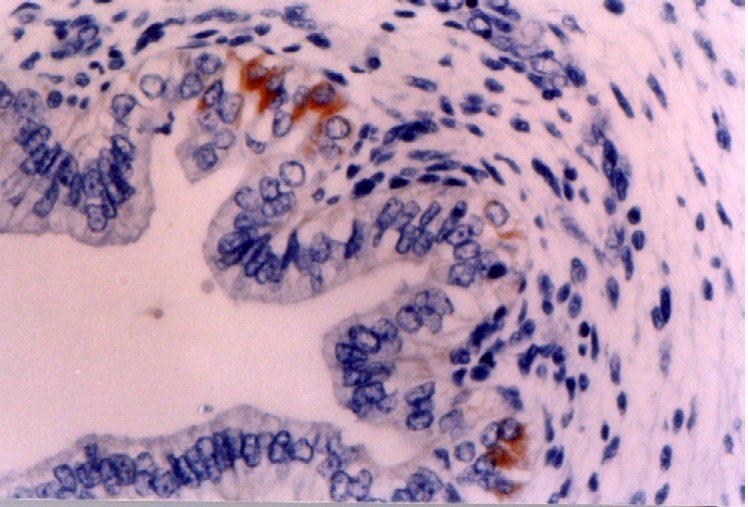
**8^th ^week of gestation (involuntary abortions)**. Ki-1 (CD30) antigen is expressed by a small number of epithelial cryptae cells. Immunohistochemical stain X 400.

**Figure 2:**
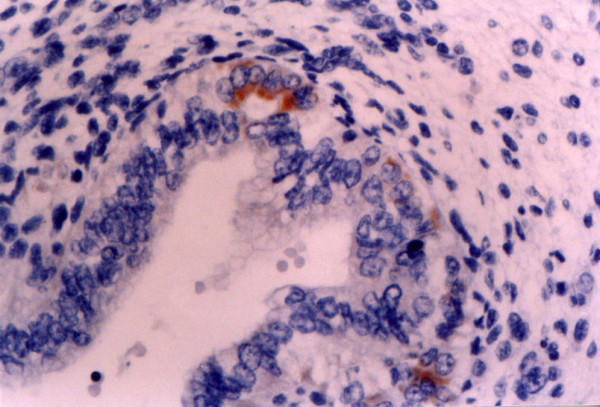
**8^th ^week of gestation (voluntary abortions)**. Weak to moderate expression of Ki-1 (CD30) antigen in the developing crypts. Immunohistochemical stain X 400.

In cases of spontaneous (involuntary) abortion, immunohistochemistry revealed small clusters or scattered, large-sized CD30/Ki-1 positive cryptae cells within the intestine in all settings examined (Fig. 1), with percentages varying from 3.2 to 3.9 (mean ± sd = 3.61 ± 0.16). In the neighbouring intestinal stroma a slight cellular infiltration was observed, consisting of rounded mononuclear cells approximately 10 μm in diameter with eccentric kidney-shaped nuclei and expressing a CD45/LCA and CD3 phenotype. In cases of voluntary or therapeutic abortion, immunohistochemistry showed a smaller number of large-sized CD30/Ki-1 positive cryptae cells in all settings examined (Fig. 2), with percentages varying from 3.1 to 3.7 (mean ± sd = 3.42 ± 0.17). No inflammatory infiltrates or necrosis were noted in the neighbouring intestinal stroma.

### 10th week of gestation

**Figure 3:**
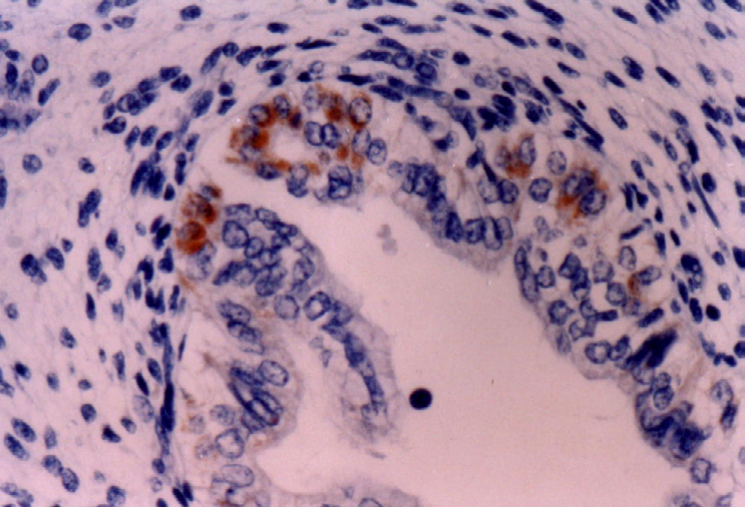
**10^th ^week of gestation (involuntary abortions)**. Strong expression of Ki-1 (CD30) antigen in the developing crypts. Immunohistochemical stain X 400

In cases of spontaneous abortion, immunohistochemistry showed a higher number of positive CD30/Ki-1 cryptae cells than at the 8th week of gestation (Fig. 3), with percentages varying from 4.9 to 5.6 (mean ± sd = 5.27 ± 0.19). There were very few inflammatory infiltrates in the intestinal stroma expressing the phenotype CD45/LCA and CD3. In cases of voluntary or therapeutic abortion, the frequency of CD30/Ki-1 positive cryptae cells was similar to that at the 8th week of gestation, with percentages varying from 3.2 to 3.9 (mean ± sd = 3.43 ± 0.18). No inflammatory infiltrates or necrosis were noted in the neighbouring intestinal stroma.

### 12th week of gestation

In spontaneous abortion cases the number of CD30/Ki-1 positive cryptae cells was even higher than at 10th week, with percentages varying from 4.8 to 5.7 (mean ± sd = 5.34 ± 0.23). The number in cases of voluntary or therapeutic abortions was more or less the same as at 8th and 10th weeks, with percentages varying from 3.2 to 3.7 (mean ± sd = 3.41 ± 0.17). No differences in immune reaction were noted in the neighbouring intestinal stroma in cases of either spontaneous or voluntary/therapeutic abortion in comparison to the 8th and 10th gestational weeks.

The differences among the numbers of CD30/Ki-1 positive cells at the 8th, 10th and 12th gestational week after spontaneous abortion were statistically significant (p < 0.0001). No significant differences were observed in the numbers of these cells after voluntary or therapeutic abortions (p = 0.95).

## Discussion

The value of the CD30 antigen as a diagnostic marker for Hodgkin's lymphoma and anaplastic large cell lymphoma is well documented [4,5,31]. However, the function of this cytokine receptor in Hodgkin's lymphoma and other CD30-positive diseases is still not clear.

CD30 appears to have an important immunoregulatory role in normal T cell development. In normal cells, this transmembrane glycoprotein can be induced on B and T lymphocytes by mitogen stimulation or viral transformation [32-34]. cDNA cloning has revealed that the CD30 protein is a cytokine receptor of the tumor necrosis factor receptor superfamily [1,35], the ligand of which belongs to the tumor necrosis factor family [22,23].

Recent in vitro data indicate that the CD30 receptor-ligand complex can mediate signals for cell proliferation, apoptosis and cytotoxicity in lymphoid cells [20,36,37]. Our results give the first indication that the CD30 antigen is expressed in the epithelial cells of developing intestinal crypts. This observation has a number of important implications. First, our findings are of significance with regard to the accepted origin of R-S cells. Care must be taken when drawing histogenetic conclusions based on the identification of a single marker in different cell types. Shared expression of CD30 antigen does not necessarily relate Hodgkin and R-S cells to activated lymphocytes. The identification of this antigen in cells as apparently disparate as activated lymphocytes, R-S cells and now human epithelial cells of the developing fetal intestinal crypts suggests that previous views about the nature of the Ki-1 antigen must be re-examined. The Hodgkin and Reed-Sternberg cells are indeed lymphocytes as they harbor rearranged immunoglobulin (in more than 90% of cases) and T cell receptors [38]. Although the expression of CD30 antigen may indicate a relationship between these cell types, it is likely to be less straightforward than was previously supposed. Identification of the normal physiological role of CD30 antigen is thus made even more imperative if these relationships are to be understood.

Second, these findings indicate that outside the lymphatic system, CD30 antigen expression in the epithelial cells of developing intestinal crypts can mediate signals for cell proliferation and differentiation in a region where other cell types (stem, goblet, Paneth and enteroendocrine) grow throughout life.

Third, CD30 expression in the epithelial cells of the developing intestinal crypts is induced by progesterone. This is a novel mechanism of CD30 induction, distinct from neoplastic transformation and viral infection of lymphocytes. The demonstration of large R-S like cells in the developing crypts within a lymphoplasmacytic infiltrate, in the same way that similar R-S like cells are observed in reactive lymph nodes, especially within the parafollicular areas, is evidence that such cells might represent the physiological counterparts of R-S cells.

The possibility that CD30 is an oncofetal antigen is supported by our positive findings in fetal intestinal cryptae cells. We have so far been able to investigate only a single tissue from a small number of fetuses of early gestational age. Pallesen and Hamilton-Dutoit [16] examined CD30 expression in normal adult, neonatal and fetal (week 28) testes, as well as other tissues (brain, spinal cord, lung, gut, kidney, erythropoietic tissue, muscle, bone and connective tissue) from fetuses of 11 and 12 weeks gestational age, with negative results. This is the first demonstration of CD30 in epithelial cells in fetal tissue. Although our results require confirmation from frozen sections, they – together with a reported positive staining in placenta [24,25] – suggest that the antigen is expressed by proliferating and differentiating epithelial cells of other than lymphoid origin. Clearly the extent of expression of CD30 antigen in embryonal tissues warrants further investigation.

## References

[B1] Durkop ABC, Latza U, Hummel M, Eitelbach F, Seed B, Stein H (1992). Molecular cloning and expression of a new member of the nerve growth factor receptor family that is characteristic for Hodgkin's disease. Cell.

[B2] Smith CA, Gruss HJ, Davis T, Anderson D, Farrah T, Baker E, Sutherland GR, Brannan CI, Copeland NG, Jenkins NA, Grabstein KH, Gliniak B, McAlister IB, Fanslow W, Alderson M, Falk B, Gimpel S, Gillis S, Din WS, Goodwin RG, Armitage RJ (1993). CD30 antigen, a marker for Hodgkin's lymphoma, is a receptor whose ligand defines an emerging family of cytokines with homology to TNF. Cell.

[B3] Smith CA, Farrah T, Goodwin RG (1994). The TNF receptor superfamily of cellular and viral proteins: activation, costimulation, and death. Cell.

[B4] Schwab U, Stein H, Gerdes J, Lemke H, Kirchner J, Schaadt M, Diehl V (1982). Production of a monoclonal antibody specific for Hodgkin and Sternberg-Reed cells of Hodgkin's disease and a subset of normal lymphoid cells. Nature.

[B5] Stein H, Mason DY, Gerdes J, O'Connor N, Wainscoat J, Pallesen G, Gatter K, Falini B, Delsol G, Lemke H, Schwarting R, Lennert K (1985). The expression of the Hodgkin's disease-associated antigen Ki-1 in reactive and neoplastic lymphoid tissue: evidence that Reed-Sternberg cells and histiocytic malignancies are derived from activated lymphoid cells. Blood.

[B6] Ellis TN, Simms PE, Slivnick DJ, Jack H, Fisher RI (1993). CD30 is a signal-transducing molecule that defines a subset of human CD45RO+ T-cells. J Immunol.

[B7] Nakamura T, Lee RK, Nam SY, Al-Ramadi BK, Koni PA, Bottomly K, Podack ER, Flavell RA (1997). Reciprocal regulation of CD30 expression on CD4+ T cells by IL-4 and IFN-γ. J Immunol.

[B8] Annunziato F, Manetti R, Cosmi L, Galli G, Heusser CH, Romagnani S, Maggi E (1997). Opposite role for interleukin-4 and inteferon-gamma on CD30 and lymphocyte activation gene-3 (LAG-3) expression by activated naïve T cells. Eur J Immunol.

[B9] Gilfillan MC, Noel PJ, Podack ER, Reiner SL, Thompson CB (1998). Expression of the costimulatory receptor CD30 is regulated by both CD28 and cytokines. J Immunol.

[B10] Gruss HJ, Dower SK (1995). Tumor necrosis ligand superfamily: involvement in the pathology of malignant lymphomas. Blood.

[B11] Del Prete G, De Carli M, Almerigogna F, Daniel CK, D' Elios MM, Zanguoghi G, Vinante F, Pizzolo G, Romagnani S (1995). Preferential expression of CD30 by human CD4+ T cells producing Th2-type cytokines. FASEB J.

[B12] Manetti R, Annunziato F, Biagiotti R, Giudizi MG, Piccini MP, Giannarini L, Sampognaro S, Parronichi P, Vinante F, Pizzolo G, Maggi E, Romagnani S (1994). CD30 expression by CD8+ T cells producing type 2 helper cytokines: Evidence for large numbers of CD8+ CD30+ T cell clones in human immunodeficiency virus infection. J Exp Med.

[B13] Bengtsson A, Hohansson C, Linder MT, Hallden G, van der Ploeg I, Scheynius A (1995). Not only Th2 cells but also Th1 and Th0 cells express CD30 after activation. J Leukoc Biol.

[B14] Hamann D, Hilkens CM, Grogan JL, Lens SM, Kapsenberg ML, Yazdanbakhsh M, van Lier RA (1996). CD30 expression does not discriminate between human Th1- and Th2-type T cells. J Immunol.

[B15] Romagnani P, Annuziatoa F, Manetti R, Mavilia C, Lasagni L, Manuelli C, Vanelli V, Maggi E, Pupilli C, Romagnani S (1998). High CD30 ligand expression by epithelial cells and Hassal's corpuscles in the medulla of the thymus. Blood.

[B16] Pallesen G, Hamilton-Dutoit SJ (1988). Ki-1 (CD30) antigen is regularly expressed by tumor cells of embryonal carcinoma. Am J Pathol.

[B17] Pallesen G (1990). The diagnostic significance of the CD30 (Ki-1) antigen. Histopathology.

[B18] Ferreiro JA (1994). Ber-H2 expression in testicular germ cell tumors. Hum Pathol.

[B19] De Peralta-Venturina MN, Ro JY, Ordonez NG, Ayala AG (1994). Diffuse embryoma of the testis, an immunohistological study of two cases. Am J Clin Pathol.

[B20] Latza U, Foss HD, Durkop H, Eitelbach F, Dieckmann KP, Loy V, Unger M, Pizzolo G, Stein H (1995). CD30 antigen in embryonal carcinoma and embryogenesis and release of the soluble molecule. Am J Pathol.

[B21] Hittmair A, Rogatsch H, Hobisch A, Mikuz G, Feichtinger H (1996). CD30 expression in seminoma. Hum Pathol.

[B22] Suster S, Moran CA, Domoguez-Malagon H, Quevedo-Blanco P (1998). Germ cell tumors of the mediastinum and testis: a comparative immunohistochemical study of 120 cases. Hum Pathol.

[B23] Mechtesheimer G, Moller P (1990). Expression of Ki-1 antigen (CD30) in mesenchymal tumors. Cancer.

[B24] Ito K, Watanabe T, Horie R, Shiota M, Kawamura S, Mori S (1994). High expression of the CD30 molecule in human decidual cells. Am J Pathol.

[B25] Papadopoulos N, Galagios G, Anastasiadis P, Kotini A, Stellos K, Petrakis G, Zografou G, Polihronidis A, Tamiolakis D (2001). Human decidual cells can express the Hodgkin's cell-associated antigen Ki-1 (CD 30) in spontaneous abortions during the first trimester of gestation. Clin Exp Obstet Gynecol.

[B26] Sohail D, Simpson RH (1991). Ber-H2 staining of lipoblasts. Histopathology.

[B27] Mechtesheimer G, Kruger KH, Born IA, Moller P (1990). Antigenic profile of mammary fibroadenoma and cystosarcoma phyllodes. A study using antibodies to estrogen- and progesterone receptors and to a panel of cell surface molecules. Pathol Res Pract.

[B28] Rudolph P, Lappe T, Schmidt D (1993). Expression of CD30 and nerve growth factor-receptor in neoplastic and reactive vascular lesions: an immunohistochemical study. Histopathology.

[B29] Garcia-Prats MD, Ballestin C, Sotelo T, Lopez-Encuentra A, Mayordomo JI (1998). A comparative evaluation of immunohostochemical markers for the differential diagnosis of malignant pleural tumours. Histopathology.

[B30] Anderssen R, Brugger W, Lohr GW, Bross KJ (1989). Human macrophages can express the Hodgkin's cell-associated antigen Ki-1 (CD30). Am J Pathol.

[B31] Schwarting R, Gerdes J, Dürkop H, Falini B, Pireli S, Stein H (1989). Ber-H2, a new anti-Ki-1 (CD30) monoclonal antibody directed at a formol-recistant epitope. Blood.

[B32] Stein H, Gerdes J, Schwab U, Lemke H, Mason DY, Ziegler A, Schienle W, Diehl V (1982). Intentification of Hodgkin and Sternberg-Reed cells as a unique cell type derived from a newly-detected small cell population. Int J Cancer.

[B33] Froese P, Lemke H, Gerdes J, Havensteen B, Schwarting R, Hansen H, Stein H (1987). Biochemical characterization and biosynthesis of the Ki-1 antigen in Hodgkin-derived and virus-transformed human B and T lymphoid cell lines. J Immunol.

[B34] Andreesen R, Osterholz J, Lohr GW, Bross KJ (1984). A Hodgkin cell-specific antigen is expressed on a subset of auto- and alloactivated T (helper) lymphoblasts. Blood.

[B35] Mallet S, Barclay AN (1991). A new superfamily of cell surface proteins related to the nerve growth factor receptor. Immunol Today.

[B36] Beutler B, van Huffel C (1994). Ultraveling fuction in the TNF ligand and receptor families. Science.

[B37] Bowen MA, Olsen KJ, Lirong Cheng, Avila D, Rodack ER (1993). Fuctional effects of CD30 on a large granular lymphoma cell line. YT. J Immunol.

[B38] Kadin M (2000). Regulation of CD30 antigen expression and its potential significance for human disease. Am J Pathol.

